# Excitation-inhibition balance and auditory multistable perception are correlated with autistic traits and schizotypy in a non-clinical population

**DOI:** 10.1038/s41598-020-65126-6

**Published:** 2020-05-18

**Authors:** Hirohito M. Kondo, I-Fan Lin

**Affiliations:** 1 School of Psychology, Chukyo University, Nagoya, Aichi 466-8666 Japan; 2 Human Information Science Laboratory, NTT Communication Science Laboratories, NTT Corporation, Atsugi, Kanagawa 243-0198 Japan; 3 Department of Occupational Medicine, Shuang Ho Hospital, New Taipei City, 235 Taiwan; 4 Department of Medicine, Taipei Medical University, Taipei, 110 Taiwan

**Keywords:** Perception, Human behaviour

## Abstract

Individuals with autism spectrum disorder and individuals with schizophrenia have impaired social and communication skills. They also have altered auditory perception. This study investigated autistic traits and schizotypy in a non-clinical population as well as the excitation-inhibition (EI) balance in different brain regions and their auditory multistable perception. Thirty-four healthy participants were assessed by the Autism-Spectrum Quotient (AQ) and Schizotypal Personality Questionnaire (SPQ). The EI balance was evaluated by measuring the resting-state concentrations of glutamate-glutamine (Glx) and ϒ-aminobutyric acid (GABA) *in vivo* by using magnetic resonance spectroscopy. To observe the correlation between their traits and perception, we conducted an auditory streaming task and a verbal transformation task, in which participants reported spontaneous perceptual switching while listening to a sound sequence. Their AQ and SPQ scores were positively correlated with the Glx/GABA ratio in the auditory cortex but not in the frontal areas. These scores were negatively correlated with the number of perceptual switches in the verbal transformation task but not in the auditory streaming task. Our results suggest that the EI balance in the auditory cortex and the perceptual formation of speech are involved in autistic traits and schizotypy.

## Introduction

Sensory abnormalities are a widely known but poorly understood character of autism spectrum disorder (ASD) and schizophrenia. Previous studies have shown that the brains of individuals with ASD and individuals with schizophrenia may have an atypical excitation-inhibition (EI) balance, which is region-specific and age-specific^1^. The main excitation and inhibition neurotransmitters in the brain are glutamate and ϒ-aminobutyric acid (GABA), respectively. The disruption of glutamate-mediated excitation and GABA-mediated inhibition would affect brain function^2^. If the EI balance in the neural systems is disrupted, sensory processing could be affected. Specifically, losing excitation control could reduce sensory stimulation, and losing inhibitory control could cause the neural system to be overwhelmed by stimulation. To ensure a dynamic yet robust system, the balance between excitatory and inhibitory networks must be maintained over time.

After our sensory organs receive inputs from the environment, it is important for our neural systems to process these input signals to construct meaningful perceptual objects. In auditory perception, the integration and segregation of sequential acoustic signals play an important role in auditory scene analysis^3^. When we listen to an auditory sequence with repeated units, we may switch our perceptions because the acoustic components inside the sequence are integrated and segregated differently over time. This is known as multistable perception, which provides a clue to probing auditory scene analysis because physically unchanging stimulation leads to spontaneous switching between different stable percepts. The present study used the number of perceptual switches in auditory streaming and verbal transformations as an index of the perceptual characteristics for each of the participants. We could also identify individual differences in terms of sensitivity to speech sounds when we compared their responses in the auditory streaming task and in the verbal transformation task because the former contained a sequence of pure tones, and the latter contained a sequence of speech sounds.

Our previous study, which used magnetic resonance spectroscopy (MRS), measured the concentration of glutamate-glutamine (Glx) and GABA within brain regions and investigated the correlation of Glx and GABA and auditory multistable perception^4^. We found a positive correlation between Glx in the auditory cortex (AC) and the proportion of segregated percepts in auditory streaming and the duration of percepts for the word “banana” in verbal transformations. In addition, we found a negative correlation between the concentration of GABA in the inferior frontal cortex (IFC) and the duration of a segregated percept in auditory streaming and duration of other percepts in verbal transformations. Glx and GABA concentrations in the AC and IFC are involved in the formation and selection of auditory objects, and, therefore, an abnormal EI balance in the AC and IFC may lead to abnormal auditory perception.

Autism has been distinguished from childhood-onset psychosis since DSM-III was developed in 1980. However, a high comorbidity of early onset schizophrenia and autism spectrum disorder (ASD) has been noted^5^. Individuals in these two groups have also been reported to have abnormal sensory perception, such as auditory hypersensitivity in ASD and hallucination in schizophrenia. In previous studies using multistability paradigms to investigate the auditory perception of the two groups, individuals with ASD were found to have the reduction of electrophysiological responses for auditory stream segregation^6^, and schizophrenic patients were found to be less sensitive to the effect of frequency separation in auditory stream segregation^7^. In a verbal transformation experiment, the number of perceptual switches for schizophrenic patients with auditory hallucinations was similar to that for matched healthy controls^8^, and individuals with ASD were found to perceive more drastic changes for the same sequence of speech sounds^9^. These findings have been rather mixed, but the sensitivity to auditory multistability in ASD and schizophrenic groups probably differs in the auditory streaming task and in the verbal transformation task.

**Figure 1:**
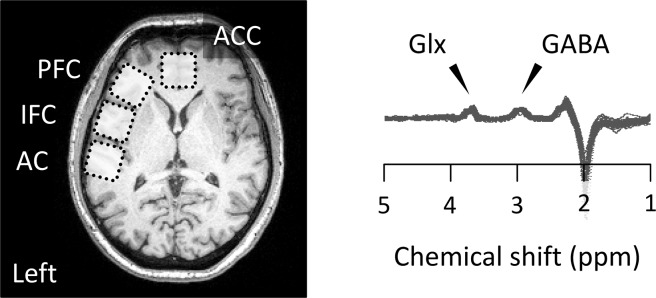
Size and location of voxels and edited MR spectra (*N* = 34). MRS voxels are positioned in auditory cortex (AC), inferior frontal cortex (IFC), prefrontal cortex (PFC) and anterior cingulate cortex (ACC). Glx and GABA peaks were calculated using differences between spectra obtained by editing radio frequency on/off pulses. GABA, γ-aminobutyric acid; Glx, glutamate-glutamine.

In this study, we recruited thirty-four non-clinical participants and examined the correlation between their autistic traits and schizotypy, auditory multistable perception and EI balance in their brain. Although the Autism-Spectrum Quotient (AQ) is not a standard clinical tool for ASD diagnosis, it is used as a screening tool and reveals hereditary properties^10^. Schizotypy, assessed by the Schizotypal Personality Questionnaire (SPQ), is found to overlap with schizophrenia, structure-wise, function-wise and neurotransmitter-wise^11^. To clarify low- and high-level brain functions, we focused on the EI balance in the AC and IFC as well as in the prefrontal cortex (PFC) and anterior cingulate cortex (ACC) (Fig. 1). We hypothesized that participants with autistic traits and participants with schizotypy would have an increased EI balance based on the hypersensitivity in ASD and auditory hallucination in schizophrenia. On the basis of previous findings, we tested the hypothesis that participants with autistic traits and participants with schizotypy have a reduced number of perceptual switches in auditory multistable perception. In addition, we used structural equation modelling (SEM) to discuss which kind of relationship can explain autistic and schizotypal traits. We postulated that these two traits have different origins but share the same vulnerabilities.

## Results

### Descriptive statistics of AQ and SPQ scores

The total and subscores of the AQ and SPQ are shown in Table 1. For the AQ scores, we computed scores of two factors, one corresponding to the attention to detail score (AQ Detail) and another obtained by adding together the remaining scores (AQ Interpersonal). Cronbach’s alpha of AQ and SPQ scores reached a satisfactory level (range 0.73 to 0.95), suggesting that these scores have a high level of internal consistency. The skewness and kurtosis of the data distribution were 0.19 and 0.59 for the AQ total scores and 1.01 and 0.41 for the SPQ total scores. Using Kolmogorov–Smirnov tests, we identified that the AQ total scores followed a normal distribution (*p* > 0.20), but the SPQ total scores did not (*p* = 0.080) (Supplementary Figure 1). Three participants had an AQ total score of more than 32, which suggests a cut-off score for a clinically significant level of autistic traits^12^. Four participants had an SPQ total score of more than 38, which indicates a cut-off score for the assessment of possible schizotypal personality^13^. The AQ total scores (mean ± standard error) were greater for males (24.0 ± 1.9) than for females (15.6 ± 1.9): *t* = 2.81, *p* = 0.008, Cohen’s *d* = 1.06. In comparison, the SPQ total scores did not differ between males (20.5 ± 3.1) and females (15.7 ± 3.8): *t* = 0.92, *p* > 0.36, Cohen’s *d* = 0.35. Advancing age led to a decrease in the AQ and SPQ total scores: *r* = −0.36, *p* = 0.037; *r* = −0.27, *p* > 0.12.

**Table 1:** Descriptive statistics for Autism-Spectrum Quotient (AQ) and Schizotypal Personality Questionnaire (SPQ) scores. Reliability is calculated by using Cronbach’s alpha.

Measure	Variable	Mean	SD	Min	Max	Reliability
AQ	Total	21.3	8.9	5	39	0.85
	Interpersonal	16.6	8.7	1	33	0.90
	Attention to Detail	4.7	2.6	0	10	0.73
SPQ	Total	18.9	13.8	0	55	0.95
	Cognitive-Perceptual	6.4	5.6	0	22	0.88
	Interpersonal	9.4	8.5	0	31	0.94
	Disorganized	5.0	4.1	0	14	0.86

### Correlations between AQ and SPQ scores

We found a strong positive correlation between the AQ and SPQ total scores: *r* = 0.68, *p* < 0.001 (Table 2). The AQ total scores were positively correlated with the SPQ Interpersonal subscore (*r* = 0.74, *p* < 0.001) and with the SPQ Disorganized subscore (*r* = 0.56, *p* = 0.001). The same pattern was found in the relationship between the AQ Interpersonal and SPQ Interpersonal scores (*r* = 0.75, *p* < 0.001) and between the AQ Interpersonal and SPQ Disorganized scores (*r* = 0.56, *p* = 0.001). The correlation coefficient was greater for the former than for the latter: *t* = 2.00, *p* = 0.054. However, there was no significant correlation between the AQ Detail factor and SPQ subscores. These results suggest that the general overlap between the AQ and SPQ scores was derived from the interpersonal factor of each participant.

**Table 2:** Correlations between AQ and SPQ scores. Values indicated in bold are significant after false discovery rate (FDR) correction. Cog-Per, cognitive-perceptual; Detail, attention to detail; Disorg, disorganized; Inter, interpersonal.

Measure	AQ Total	AQ Inter	AQ Detail
SPQ Total	***r*** = **0.683**	***r*** = **0.660**	*r* = 0.125
	***p*** < **0.001**	***p*** < **0.001**	*p* = 0.480
SPQ Cog-Per	*r* = 0.343	*r* = 0.261	*r* = 0.303
	*p* = 0.047	*p* = 0.137	*p* = 0.081
SPQ Inter	***r*** = **0.737**	***r*** = **0.747**	*r* = 0.021
	***p*** < **0.001**	***p*** < **0.001**	*p* = 0.907
SPQ Disorg	***r*** = **0.562**	***r*** = **0.555**	*r* = 0.064
	***p*** = **0.001**	***p*** = **0.001**	*p* = 0.721

### Relations between personality, perceptual multistability and EI balance

**Figure 2:**
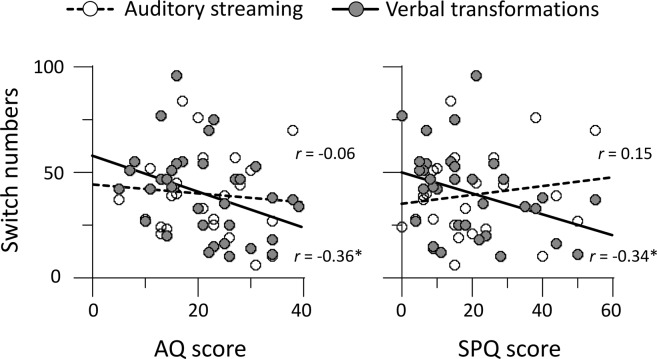
Scatter plots for relationship between scores of personality traits and number of perceptual switches. Circles indicate individual data. Solid and dashed lines represent linear regression fits derived from verbal transformations and auditory streaming, respectively. *p < 0.05.

We first checked the correlations between personality and perceptual multistability (Supplementary Table 1). The number of perceptual switches in the verbal transformation task decreased as the AQ total score increased (*r* = −0.36, *p* = 0.039), but that in the auditory streaming task did not (*r* = −0.06, *p* > 0.77) (Fig. 2). Similarly, the number of perceptual switches in the verbal transformation task decreased as the SPQ total score increased (*r* = −0.34, *p* = 0.050), but that in the auditory streaming task did not (*r* = 0.15, *p* > 0.48) (Fig. 2). In summary, individuals with higher AQ scores, as well as those with higher SPQ scores, tended to experience less perceptual switching induced by speech stimuli rather than by non-speech stimuli.

**Figure 3:**
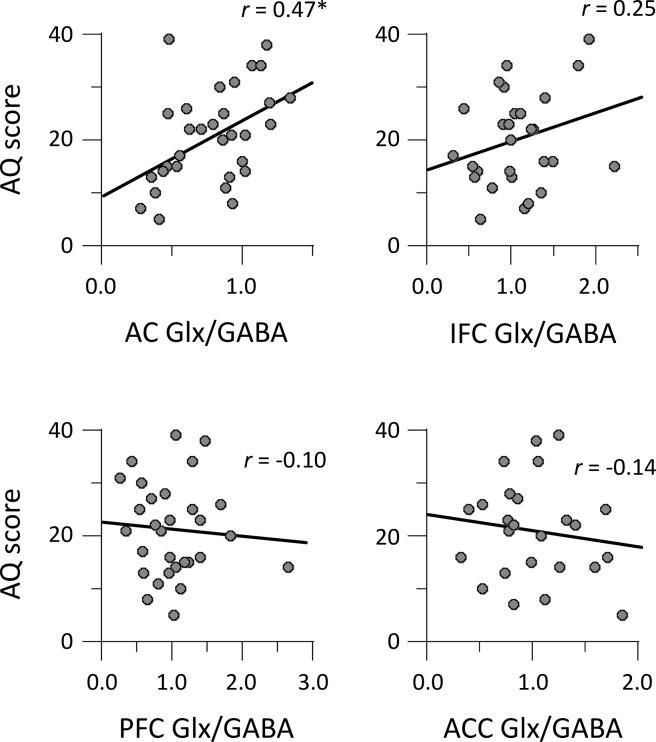
Correlations between AQ total scores and Glx/GABA ratios of voxels. AC, auditory cortex; ACC, anterior cingulate cortex; IFC, inferior frontal cortex; PFC, prefrontal cortex. *p < 0.05.

**Figure 4:**
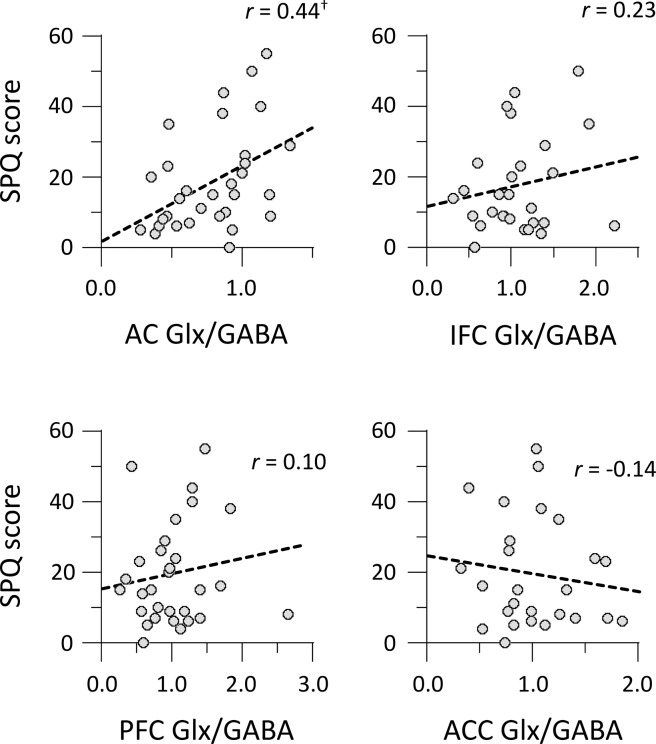
Correlations between SPQ total scores and Glx/GABA ratios of voxels. ^†^*p* < 0.10.

Next, we examined whether the AQ and SPQ total scores were correlated with the Glx/GABA ratios in four voxels. The AQ total scores were correlated with the Glx/GABA ratio in the AC (*r* = 0.47, *p* = 0.008) but were not correlated with those in the other voxels (|*r* | < 0.25, *p* > 0.20) (Fig. 3). There was a significant correlation between the SPQ total score and Glx/GABA ratio in the AC: *r* = 0.44, *p* = 0.014 (Fig. 4). The correlation between the SPQ total score and Glx/GABA ratio in the other voxels did not reach statistical significance: |*r* | < 0.23, *p* > 0.23. In summary, the EI balance observed in the AC was found to be correlated with both autistic traits and schizotypy.

### Common factors underlying AQ and SPQ scores

The commonality of AQ and SPQ scores mainly relied on the interpersonal factor. This factor could be derived from the dysfunctional communication that occurs when exchanging information and meaning between two or more people. Furthermore, correlation analyses demonstrated that individuals with autistic traits and schizotypy shared common patterns in terms of perceptual changes and EI balance in the AC. These results raise the question of how participant’s personality, auditory perception and neurochemical measures are interdependent with each other.

**Figure 5:**
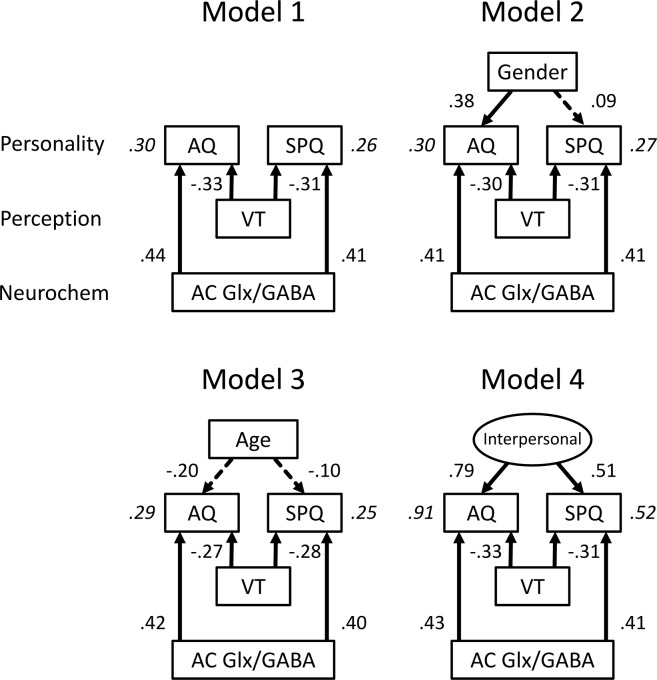
Comparisons of path diagrams of four models. Estimates alongside arrows represent standardized regression coefficients, corresponding to estimates of effective connectivity. Estimates in italics indicate multiple correlation coefficients. Solid lines indicate significant paths (*p* < 0.05), but dashed lines do not.

To further examine this issue, we built structural models that were fitted to the data. Figure 5 illustrates four *a priori* models that were considered. The AQ and SPQ scores were the dependent variables to be explained. Model 1 assumed that the Glx/GABA ratio in the AC and the number of verbal transformations were independent variables. The *χ*^2^ test showed that this model was significant: *χ*^2^_(2)_ = 12.10, *p* = 0.002. This means that the covariances predicted by Model 1 differed from the covariances of actual data. In addition, the estimate of the comparative fit index (CFI) of Model 1 was quite low (0.59). We constructed Model 2 to examine whether the fit indices were improved by entering the variable of gender to explain the AQ and SPQ scores. An improvement in the CFI estimate was observed, but that of the Akaike information criterion (AIC) estimate was not (Table 3). In Model 3, the variable of age was entered as a common factor, but the AIC and CFI estimates were worse than those in Models 1 and 2. In Model 4, we assumed the interpersonal factor to bridge between the AQ and SPQ scores. This factor was not an observed variable, and, thus, we entered it as a latent variable for affecting both scores. A *χ*^2^ difference test revealed that Model 4 provided a better fit than Model 2: *χ*^2^_(3)_ = 13.59, *p* = 0.004. The AIC and CFI estimates for Model 4 indicated a better fit to the data than those for the other models. In Model 4, standardized path coefficients from the EI balance in the AC to the AQ and SPQ scores (0.43 and 0.41) were greater than those from the number of verbal transformations to the AQ and SPQ scores (−0.33 and −0.31): *Z* > 2.65, *p* < 0.008. Thus, the SEM analysis suggested that the EI balance in the AC contributes to connecting autistic traits with schizotypy.

**Table 3:** Fit indices for structural models. Nonsignificant χ^2^ statistics and χ^2^/*df* < 2 represent good fit to data. Lower AIC values indicate better fit, whereas higher CFI values represent good fit. See explanations of fit indices in Data analysis section. AIC, Akaike information criterion; CFI, comparative fit index; *df*, degree of freedom.

Model	χ^2^	*df*	*p*	χ^2^*/df*	AIC	CFI
1	12.10	2	0.002	6.05	36.10	0.592
2	13.76	4	0.008	3.44	45.76	0.662
3	17.22	4	0.002	4.30	49.22	0.507
4	0.17	1	0.677	0.17	26.17	1.000

## Discussion

We measured EI balance and auditory multistable perception in a non-clinical population and evaluated the correlation between these measures and the participants’ autistic and schizotypal traits. We found significant correlations between autistic traits and schizotypy. Individuals with higher AQ or SPQ scores had a smaller number of perceptual switches in the verbal transformation task. The EI balance in the AC was positively correlated with their AQ and SPQ scores, but that in the IFC, PFC and ACC was not. These findings bring up two questions. Firstly, why are autistic and schizotypal traits associated with the number of switches in the verbal transformation task? Secondly, how can we explain the association between the EI balance in the brain and the AQ or SPQ scores? We discuss the relationship between the AQ and SPQ scores and then consider these questions in turn.

We demonstrated that the AQ total scores were associated with the SPQ total scores. This association is consistent with previous studies^14–17^. Both AQ and SPQ questionnaires include several dimensions, and we need to consider the possibility that some AQ’s subscales change in the same direction with some SPQ’s subscales, but some AQ’s subscales are the opposite of some SPQ’s subscales. For example, the AQ imagination subscale seems to be in contrast with the fluidity of thought observed in schizophrenia. In addition, the social skills and communication subscales in the AQ can be closely related to the Interpersonal factor in the SPQ. For example, despite the fact that individuals with ASD and individuals with schizophrenia may misinterpret social cues in different ways, misinterpretation of social cues always leads to defective social interpersonal communication and paranoid thoughts. Indeed, our results confirm that the overlap between the AQ and SPQ scores is derived from the interpersonal factor.

In a verbal transformation experiment, a listener listens to a stream of speech sounds, and the speech sounds could be grouped and segregated in several different ways. When the listener hears a set of a repeated meaningful or non-meaningful word, this word becomes an auditory object that is separated from the “background” and becomes the “foreground”. When the perceptual switch occurs, the previous “background” becomes a “foreground”, and the previous “foreground” becomes a “background”. In the computational model, perceptual formation and switching in multistability can be explained by the mutual inhibition between different percepts and adaptation of this inhibition^18^. On the basis of these models, a reduced GABA concentration may induce reduced mutual inhibition, and the dysfunction of mutual inhibition leads to less discrimination of received stimuli and longer processing time for the winning percept to build up. There is a study showing that individuals with schizophrenia have less lateral inhibition^19^. Our findings of the smaller number of verbal transformations with higher AQ or SPQ scores also indicate that these participants are poor at exploiting sequential information outside the focus of attention.

More importantly, we found an asymmetry in perceptual formation between speech and non-speech stimuli; the small number of perceptual switches was observed only in the verbal transformation task when the stimuli were speech sounds but not in the auditory streaming task when the stimuli were pure tones. It is known that individuals with ASD show superior performance in frequency discrimination^20^ and melody discrimination^21^. Previous studies have demonstrated that individuals with ASD have a normal involuntary orientation to pure tones and complex tones, whereas they have less or delayed involuntary orientation to speech^22–24^. The small number of switches for verbal transformations might be related to their reduced involuntary attention to speech. In contrast, individuals with schizophrenia have auditory dysfunction in another way; their performance on frequency discrimination tasks is impaired^25,26^. Their poor performance may lead to impairments in more complex skills, such as categorical perception of speech^27^ and encoding speech prosody^28^. For example, a previous study argued that the mismatch negativity in phoneme stimuli, indicating the neural coding of phonemes, reflects social skills in schizophrenia^29^. Thus, there is the possibility that perceptual formation in verbal transformations is mediated by both sensory processing and speech comprehension.

There has been controversy as to whether perceptual formation occurs in the sensory cortex or in the hierarchically high-level cortex. The global neuronal workspace theory states that consciousness is supported by distributed networks of brain regions, and attention plays an important role in perceptual formation^30–32^. This theory emphasizes the long-range connectivity bridging different systems. It has been suggested that ASD and schizophrenia disrupt large-scale brain connectivity^33^. There is also a computational model using top-down predictions and prediction errors to explain perceptual formation and switching^34^. In recurrent processing theory, feedforward and feedback (recurrent) processes between the primary and peripheral sensory areas are sufficient for consciousness^35,36^.

Perceptual switching in auditory multistability has been shown to involve a broad neural network from subcortical areas of the auditory pathway to several cortical areas, including the cochlear nucleus^37^, medial geniculate body^38^, caudate nucleus^39^, auditory cortex^40^, parietal cortex^41^ and frontal cortex^42^. We demonstrated that AQ and SPQ scores were correlated with EI balance in the AC but were not with EI balance in the frontal cortex. Thus, this finding supports the argument that the abnormality in ASD and schizophrenia may not necessarily take place in the frontal cortex or in the global connectivity, and the EI balance in the primary sensory cortex may explain why individuals with ASD and schizophrenia perceive objects differently.

Glutamic acid decarboxylase (GAD) plays a critical role in GABA production, and the downregulation of GAD is found in ASD^43,44^ and in schizophrenia^45–47^. A reduced GABA level indicates a cortical EI imbalance and a disruption in synaptic mechanisms, which then contributes to an individual’s psychopathology^48,49^. Previous MRS studies have also revealed that the GABA level in the AC was lower for ASD children than for typically developing children^50,51^. Compared with typically developing individuals, individuals with schizophrenia had similar concentrations of glutamate in Heschl’s gyrus and temporal plane^52^ but increased concentrations of GABA in the right superior temporal lobe and inferior parietal lobe^53^. We should note, that in many studies, participants with schizophrenia were under medication, and the medication may have changed the observed neurotransmitters. In a previous study, researchers also found that the dose of antipsychotics is correlated with the GABA levels in some brain regions in the tested participants^53^.

In addition to the effect of EI balance on psychiatric disorders and on auditory perception, we would like to point out the possibility that other neurotransmitters might be involved in psychiatric disorders and auditory perception. Our previous study showed that the number of perceptual switches is affected by different catechol-*O*-methyltransferase (COMT) genetic polymorphisms^54^. COMT is an enzyme regulating the metabolism of catecholamines, including dopamine and noradrenaline. There have been debates about the association between COMT genetic polymorphisms in schizophrenia^55,56^, and there is new data on the association between COMT genetic polymorphisms in ASD^57^. COMT genetic polymorphisms and the related dopaminergic system in the brain are outside the scope of this paper, but this might be a direction for future research.

Our results can be summarized as follows: (1) there was a positive correlation between the AQ and SPQ total scores, (2) the AQ and SPQ scores were negatively correlated with the number of verbal transformations, (3) these scores were positively correlated with the Glx/GABA ratio in the AC, and (4) the contribution of EI balance to the AQ and SPQ scores was greater than the contribution of the auditory perception in the verbal transformation task. Overall, these findings support our hypothesis that those with autistic traits and those with schizotypy have an increased EI balance and a reduced number of perceptual switches in speech-related multistable perception.

## Materials and Methods

### Ethics statement

This study was approved by the Ethics and Safety Committees of NTT Communication Science Laboratories and ATR-Promotions (approval nos. H28–015 and AN16–004) and carried out in accordance with the Ethical Guidelines for Medical and Health Research Involving Human Subjects. All participants gave written informed consent after the procedures had been fully explained to them.

### Participants

Thirty-four participants (23 males and 11 females; range 21–60 years; *M*_age_ = 37.4, *SD*_age_ = 10.7) were recruited for the experiments. They were right-handed with normal or corrected-to-normal vision and with normal hearing. None had any history of neurological or psychiatric disorders. After the MRS data acquisition, participants filled out personality questionnaires and performed behavioural tasks in a quiet room.

### Personality questionnaires

The AQ is used to assess autistic traits in adults of normal intelligence. The AQ consists of 50 items spanning five domains: social skills, attention switching, attention to detail, communication and imagination^12^. Participants scored the items on a 4-point Likert-scale from “definitely agree” to “definitely disagree”. The Likert-scale values were collapsed into two categories. This is the recommended scoring procedure, resulting in a maximum total score of 50. Previous studies have demonstrated that the AQ can be represented by at least two correlated factors: an attention to detail factor and interpersonal factor^14,58,59^.

Schizotypy was assessed using the SPQ, which is a 74-item questionnaire with a dichotomous response format. The SPQ was developed to measure all nine features of SPD defined by the DSM-III-R. A factor analytical study has shown that the nine diagnostic features for SPD can be integrated into three factors: Cognitive-Perceptual, Interpersonal and Disorganized^13^. The Cognitive-Perceptual factor, which reflects positive symptoms of SPD, is characterized by ideas of reference, odd beliefs/magical thinking, unusual perceptual experiences and suspiciousness/paranoid ideation. The Interpersonal factor, which reflects negative symptoms of SPD, is characterized by excessive social anxiety, no close friends, constricted affect and suspiciousness/paranoid ideation. The Disorganized factor, which is related to disorganized symptoms, consists of eccentric behaviour and odd speech. In this study, scores for all items were added together to produce the SPQ total score and the three subscores.

For a preliminary analysis, we performed a factor analysis to identify the relationship between the AQ and SPQ subscales (Supplementary Figure 2). Two factors, whose eigenvalues were larger than 1, were extracted from the eight subscales. The first factor, with an eigenvalue of 4.28 before varimax rotation, was heavily loaded on the AQ social skills, communication, imagination, attention switching and the SPQ Interpersonal scores (factor loadings, 0.70 to 0.88). The second factor, with an eigenvalue of 1.50, was loaded on the SPQ Cognitive-Perceptual and Disorganized factors and the AQ Detail scores (0.34 to 0.96). These two factors accounted for a 66.2% variance of all the AQ and SPQ subscores. Therefore, our results were generally consistent with previous findings^14,59^.

### Behavioural tasks

Participants performed auditory streaming and verbal transformation tasks separately. Stimulus presentation and response collection were managed using the Presentation software (Neurobehavioral Systems, Berkeley, CA, USA). The presentation level was set at a sound pressure level (SPL) of 70 dB. The stimuli were delivered through Sennheiser HDA 200 headphones. The test trials were preceded by training trials, where participants practiced reporting their perception confidently and precisely^4^. The number of perceptual switches was computed for each 4-min trial of auditory streaming and verbal transformations.

In the auditory streaming task, the auditory stimulus was a sequence of a repeating ABA- tone pattern, where A and B represent two pure tones at different frequencies, and the hyphen represents a silent interval. The stimulus parameters were identical to those used in previous studies^38,54^. The A and B tones were centred on 1 kHz with a four-semitone frequency difference between them (frequency for A = 891 Hz; frequency for B = 1122 Hz). The duration of each tone was 40 ms, including 10-ms rising and falling cosine ramps. The stimulus onset asynchrony between successive tones was 100 ms. Participants were instructed to report in real-time whether they heard one stream (ABA-ABA-…) with a galloping rhythm or two streams (A-A-… and -B—B—…) with an isochronous rhythm for each stream. Their responses were collected via two keys on a computer keyboard: one key for a one-stream percept, and the other key for a two-stream percept. A response indicated by a key press was held until a subsequent key press.

In the verbal transformation task, the auditory stimulus consisted of repetitions of the word “banana”, spoken by a female native Japanese speaker^42^. The duration of the word was 340 ms without gaps. The participants were asked to continuously indicate the word they heard by holding down the assigned keys. These keys represented alternatives of banana: “nappa” (“vegetables” in English), some other word (i.e., actual Japanese words other than banana and nappa) and some nonsense word (undecided).

### Imaging data acquisition

To minimize confounding factors affecting neurochemical measures, we conducted the acquisition of MR spectra at a fixed time for all participants, namely between 1:00 p.m. and 4:00 p.m. Participants were scanned on a 3-T MRI scanner (MAGNETOM Trio, Siemens) using a body coil as a transmitter and a 12-channel head coil as a receiver. To assess cortical thickness and volume, three-dimensional anatomical images of the whole brain were acquired with a T1-weighted MPRAGE sequence: repetition time (TR) = 2250 ms; echo time (TE) = 3.06 ms; inversion time = 900 ms; flip angle = 9°; matrix size = 256 × 256; isotropic voxel size of 1 mm^3^.

The MRS session consisted of four runs for the different 30 × 30 × 30 mm^3^ voxels, positioned in the AC, IFC, PFC and ACC (Fig. 1). The AC voxel included Heschl’s gyrus (Brodmann area: BA 41) and the anterior part of the temporal plane (BA 42). The IFC voxel included the pars opercularis (BA 44) and pars triangularis (BA 45) of the inferior frontal gyrus. The PFC voxel was located at the anterior part of the middle frontal gyrus (BA 46). The ACC voxel (including portions of BAs 32 and 9) was located superior to the genu of the corpus callosum and centred on the interhemispheric fissure. All the voxels, except the ACC voxel, were angled parallel to the surface of the left hemisphere. The voxels were carefully placed to exclude cerebral spinal fluid (CSF) from the ventricles.

Before each run, we carried out manual shimming (approximately 5 min) of the magnetic field in the voxels. The MEGA-PRESS technique^60^ was used to obtain GABA-edited spectra from single-voxel acquisitions: TR = 2000 ms; TE = 68 ms; 128 measurements (i.e., 64 on-off pairs); spectral bandwidth of 2 kHz with a sampling rate of 2048 points; editing pulses applied at 1.9 ppm (edit-on) and 7.5 ppm (edit-off). The MRS session lasted approximately 60 min.

### Data analysis

The MRS data were analysed using TARQUIN (version 4.2.10)^61^. The data were Fourier-transformed to a spectrum of 2048 data points and smoothed by a 3-Hz Lorentzian filter. A basis set in the software was fitted to the average spectrum enabling peak amplitudes, widths and frequencies to be optimized (Voigt function). The final results were expressed as Glx and GABA signals (peaks at 3.76 and 3.00 ppm, respectively) relative to creatine (Cr) signals^62^. Normalizing to Cr reduced the interindividual variance attributable to differences in global signal strength and CSF fraction within the voxels, yielding reliable GABA concentrations^63^. The Glx/Cr and GABA/Cr concentrations (mean ± standard error) were 0.115 ± 0.004 a.u. and 0.158 ± 0.008 a.u. for the AC, 0.118 ± 0.005 a.u. and 0.119 ± 0.007 a.u. for the IFC, 0.116 ± 0.007 a.u. and 0.129 ± 0.008 a.u. for the PFC and 0.104 ± 0.005 a.u. and 0.123 ± 0.014 a.u. for the ACC. The fit errors related to the Glx and GABA concentrations were 13.4 ± 0.6% and 9.6 ± 0.5% for the AC, 15.9 ± 0.5% and 12.7 ± 0.5% for the IFC, 15.9 ± 0.7% and 12.7 ± 0.6% for the PFC and 17.8 ± 0.8% and 11.9 ± 0.5% for the ACC. Glx/GABA ratios were computed to assess the EI balance in the voxels.

Statistical analyses were carried out with IBM SPSS Statistics and Amos (version 23) and R (version 3.5.1). The number of perceptual switches did not differ between auditory streaming (39.4 ± 3.9) and verbal transformations (42.1 ± 4.2): *t* = 0.48, *p* > 0.63, Cohen’s *d* = 0.13. The perceptual switching data followed a normal distribution: Kolmogorov–Smirnov tests, *p* > 0.20. We obtained the behavioural and neurochemical measures, computed Pearson’s correlation coefficients between the variables, and used false discovery rate (FDR) correction for multiple comparisons of correlations (*α*-level = 0.05).

We performed SEM analysis to examine the relation between the personality scores, perceptual switching numbers and neurochemical measures. SEM analysis evaluates path diagrams as structural models on the basis of *a priori* theoretical considerations. In our analysis, the variables were the AQ and SPQ scores, the number of perceptual switches, the Glx/GABA ratio in the AC, gender, age and the interpersonal factor (as a latent variable). We checked the Kaiser-Meyer-Olkin (KMO) statistic using zero-order correlations and partial correlations to test whether the variables in our dataset were adequate to correlate. The KMO statistic was 0.645, indicating that there was a common factor in the dataset because its value should be greater than 0.50 in order to proceed with a satisfactory factor analysis. A Bartlett’s test of sphericity showed that correlations between the variables were greater than those expected by chance: *χ*^2^ = 43.46, *p* < 0.001. We produced different models to examine which common factors affected the AQ and SPQ scores. The maximum likelihood estimation was used to specify the standardized regression coefficients.

We assessed the fit of each model to the data using some fit indices. The likelihood ratio test (e.g., *χ*^2^ test) is frequently used to evaluate model fitting. However, the *χ*^2^ statistic measures the badness of a fit compared with a saturated model. On the basis of a previous study^64^, we thus added other fit indices: the AIC and the CFI. These indices are considered to be relatively insensitive to small sample sizes (*N* < 150). The AIC is an absolute fit index, whereas the CFI is a noncentrality-based index. The AIC measures the complexity of an evaluated model in terms of the degree of freedom and penalizes more complex models. A lower AIC estimate reflects a better-fitting model. The CFI quantifies the extent to which an evaluated model is better than a null model (i.e., the worst possible model), in which all covariances between variables are set to zero. A higher CFI estimate (more than 0.90) indicates a good fit. Finally, *χ*^2^ difference tests were performed to directly compare the fits between the full and nested models. In this test, the *χ*^2^ statistic for the nested model was subtracted from the *χ*^2^ statistic for the full model; the degree of freedom was calculated with an analogue subtraction.

## Supplementary information

Supplementary Information.

## Data Availability

Data will be made available on request.

## References

[1] Foss-Feig JH (2017). Searching for cross-diagnostic convergence: neural mechanisms governing excitation and inhibition balance in schizophrenia and autism spectrum disorders. Biol. Psychiatry.

[2] Rubenstein JLR, Merzenich MM (2003). Model of autism: increased ratio of excitation/inhibition in key neural systems. Genes Brain Behav..

[3] Kondo HM, van Loon AM, Kawahara JI, Moore BCJ (2017). Auditory and visual scene analysis: an overview. Philos. Trans. R. Soc. B..

[4] Kondo HM, Farkas D, Denham SL, Asai T, Winkler I (2017). Auditory multistability and neurotransmitter concentrations in the human brain. Philos. Trans. R. Soc. B..

[5] Rapoport J, Chavez A, Greenstein D, Addington A, Gogtay N (2009). Autism spectrum disorders and childhood-onset schizophrenia: clinical and biological contributions to a relation revisited. J. Am. Acad. Child Adolesc. Psychiatry.

[6] Lepistö T (2009). Auditory stream segregation in children with Asperger syndrome. Biol. Psychol..

[7] Weintraub DM (2012). Auditory stream segregation impairments in schizophrenia. Psychophysiology.

[8] Haddock G, Slade PD, Bentall RP (1995). Auditory hallucinations and the verbal transformation effect: the role of suggestions. Pers. Individ. Differ..

[9] Itoi C, Kato N, Kashino M (2019). People with autism perceive drastic illusory changes for repeated verbal stimuli. Sci. Rep..

[10] Wheelwright S, Auyeung B, Allison C, Baron-Cohen S (2010). Defining the broader, medium and narrow autism phenotype among parents using the Autism Spectrum Quotient (AQ). Mol. Autism.

[11] Ettinger U, Meyhofer I, Steffens M, Wagner M, Koutsouleris N (2014). Genetics, cognition, and neurobiology of schizotypal personality: a review of the overlap with schizophrenia. Front Psychiatry.

[12] Baron-Cohen S, Wheelwright S, Skinner R, Martin J, Clubley E (2001). The autism-spectrum quotient (AQ): evidence from Asperger syndrome/high-functioning autism, males and females, scientists and mathematicians. J. Autism Dev. Disord..

[13] Raine A (1994). Cognitive-perceptual, interpersonal, and disorganized features of schizotypal personality. Schizophr. Bull..

[14] Hurst RM, Nelson-Gray RO, Mitchell JT, Kwapil TR (2007). The relationship of Asperger’s characteristics and schizotypal personality traits in a non-clinical adult sample. J. Autism Dev. Disord..

[15] Dinsdale NL, Hurd PL, Wakabayashi A, Elliot M, Crespi BJ (2013). How are autism and schizotypy related? Evidence from a non-clinical population. PLoS One.

[16] Ford TC, Nibbs R, Crewther DP (2017). Increased glutamate/GABA+ ratio in a shared autistic and schizotypal trait phenotype termed Social Disorganisation. Neuroimage Clin..

[17] Ford TC, Nibbs R, Crewther DP (2017). Glutamate/GABA+ ratio is associated with the psychosocial domain of autistic and schizotypal traits. PLoS One.

[18] Noest AJ, van Ee R, Nijs MM, van Wezel RJA (2007). Percept-choice sequences driven by interrupted ambiguous stimuli: a low-level neural model. J. Vis..

[19] Ramage EM (2015). Preliminary evidence for reduced auditory lateral suppression in schizophrenia. Schizophr. Res..

[20] Bonnel A (2010). Enhanced pure-tone pitch discrimination among persons with autism but not Asperger syndrome. Neuropsychologia.

[21] Mottron L, Peretz I, Ménard E (2000). Local and global processing of music in high-functioning persons with autism: beyond central coherence?. J. Child Psychol. Psychiatry.

[22] Čeponienė R (2003). Speech-sound-selective auditory impairment in children with autism: they can perceive but do not attend. Proc. Natl. Acad. Sci. USA.

[23] Kasai K (2005). Delayed automatic detection of change in speech sounds in adults with autism: a magnetoencephalographic study. Clin. Neurophysiol..

[24] Lepistö T (2005). The discrimination of and orienting to speech and non-speech sounds in children with autism. Brain Res..

[25] Javitt DC, Shelley A-M, Ritter W (2000). Associated deficits in mismatch negativity generation and tone matching in schizophrenia. Clin. Neurophysiol..

[26] Rabinowicz EF, Silipo G, Goldman R, Javitt DC (2000). Auditory sensory dysfunction in schizophrenia: imprecision or distractibility?. Arch. Gen. Psychiatry.

[27] Cienfuegos A, March L, Shelley A-M, Javitt DC (1999). Impaired categorical perception of synthetic speech sounds in schizophrenia. Biol. Psychiatry.

[28] Leitman DI (2005). Sensory contributions to impaired prosodic processing in schizophrenia. Biol. Psychiatry.

[29] Kawakubo Y (2007). Phonetic mismatch negativity predicts social skills acquisition in schizophrenia. Psychiatry Res..

[30] Dehaene, S., Kerszberg, M. & Changeux, J. P. A neuronal model of a global workspace in effortful cognitive tasks. *Proc. Natl. Acad. Sci. USA* **95**, 14529-14534, 10.1073/pnas.95.24.14529 (1998). 10.1073/pnas.95.24.14529 PMC24407 9826734

[31] Dehaene S, Naccache L (2001). Towards a cognitive neuroscience of consciousness: basic evidence and a workspace framework. Cognition.

[32] Dehaene S, Changeux JP, Naccache L, Sackur J, Sergent C (2006). Conscious, preconscious, and subliminal processing: a testable taxonomy. Trends Cogn. Sci..

[33] Wallace R (2005). A Global Workspace perspective on mental disorders. Theor. Biol. Med. Model..

[34] Hohwy J, Roepstorff A, Friston K (2008). Predictive coding explains binocular rivalry: an epistemological review. Cognition.

[35] Lamme VA (2006). Towards a true neural stance on consciousness. Trends Cogn. Sci..

[36] Lamme VA (2010). How neuroscience will change our view on consciousness. Cogn. Neurosci..

[37] Pressnitzer D, Sayles M, Micheyl C, Winter IM (2008). Perceptual organization of sound begins in the auditory periphery. Curr. Biol..

[38] Kondo HM, Kashino M (2009). Involvement of the thalamocortical loop in the spontaneous switching of percepts in auditory streaming. J. Neurosci..

[39] Kashino M, Kondo HM (2012). Functional brain networks underlying perceptual switching: auditory streaming and verbal transformations. Philos. Trans. R. Soc. B..

[40] Micheyl C, Tian B, Carlyon RP, Rauschecker JP (2005). Perceptual organization of tone sequences in the auditory cortex of awake macaques. Neuron.

[41] Kondo HM, Pressnitzer D, Shimada Y, Kochiyama T, Kashino M (2018). Inhibition-excitation balance in the parietal cortex modulates volitional control for auditory and visual multistability. Sci. Rep..

[42] Kondo HM, Kashino M (2007). Neural mechanisms of auditory awareness underlying verbal transformations. Neuroimage.

[43] Fatemi SH (2002). Glutamic acid decarboxylase 65 and 67 kDa proteins are reduced in autistic parietal and cerebellar cortices. Biol. Psychiatry.

[44] Fatemi SH, Reutiman TJ, Folsom TD, Thuras PD (2009). GABA_A_ receptor downregulation in brains of subjects with autism. J. Autism Dev. Disord..

[45] Bird ED (1977). Increased brain dopamine and reduced glutamic acid decarboxylase and choline acetyl transferase activity in schizophrenia and related psychoses. Lancet.

[46] Fatemi SH, Stary JM, Earle JA, Araghi-Niknam M, Eagan E (2005). GABAergic dysfunction in schizophrenia and mood disorders as reflected by decreased levels of glutamic acid decarboxylase 65 and 67 kDa and Reelin proteins in cerebellum. Schizophr. Res..

[47] Guidotti A (2000). Decrease in reelin and glutamic acid decarboxylase_67_ (GAD_67_) expression in schizophrenia and bipolar disorder: a postmortem brain study. Arch. Gen. Psychiatry.

[48] Rojas DC (2011). Transient and steady-state auditory gamma-band responses in first-degree relatives of people with autism spectrum disorder. J. Autism Dev. Disord..

[49] Parellada M (2014). The neurobiology of autism spectrum disorders. Eur. Psychiatry.

[50] Gaetz W (2014). GABA estimation in the brains of children on the autism spectrum: measurement precision and regional cortical variation. Neuroimage.

[51] Rojas DC, Singel D, Steinmetz S, Hepburn S, Brown MS (2014). Decreased left perisylvian GABA concentration in children with autism and unaffected siblings. Neuroimage.

[52] Atagün Mİ (2015). Investigation of Heschl’s gyrus and planum temporale in patients with schizophrenia and bipolar disorder: a proton magnetic resonance spectroscopy study. Schizophr. Res..

[53] Atagün Mİ (2017). Perisylvian GABA levels in schizophrenia and bipolar disorder. Neurosci. Lett..

[54] Kondo HM (2012). Separability and commonality of auditory and visual bistable perception. Cereb. Cortex.

[55] Shifman S (2002). A highly significant association between a COMT haplotype and schizophrenia. Am. J. Hum. Genet..

[56] Munafò MR, Bowes L, Clark TG, Flint J (2005). Lack of association of the *COMT* (Val^158/108^ Met) gene and schizophrenia: a meta-analysis of case-control studies. Mol. Psychiatry.

[57] Esmaiel NN (2020). The potential impact of COMT gene variants on dopamine regulation and phenotypic traits of ASD patients. Behav. Brain Res..

[58] Hoekstra RA, Bartels M, Cath DC, Boomsma DI (2008). Factor structure, reliability and criterion validity of the Autism-Spectrum Quotient (AQ): a study in Dutch population and patient groups. J. Autism Dev. Disord..

[59] Del Giudice M, Angeleri R, Brizio A, Elena MR (2010). The evolution of autistic-like and schizotypal traits: a sexual selection hypothesis. Front. Psychol..

[60] Mescher, M., Merkle, H., Kirsch, J., Garwood, M. & Gruetter, R. Simultaneous *in vivo* spectral editing and water suppression. *NMR Biomed*. **11**, 266–272, 10.1002/(SICI)1099-1492(199810)11:6<266::AID-NBM530>3.0.CO;2-J (1998). 10.1002/(sici)1099-1492(199810)11:6<266::aid-nbm530>3.0.co;2-j 9802468

[61] Wilson M, Reynolds G, Kauppinen RA, Arvanitis TN, Peet AC (2011). A constrained least-squares approach to the automated quantitation of *in vivo*^1^H magnetic resonance spectroscopy data. Magn. Reson. Med..

[62] Kihara K, Kondo HM, Kawahara JI (2016). Differential contributions of GABA concentration in frontal and parietal regions to individual differences in attentional blink. J. Neurosci..

[63] Bogner W (2010). *In vivo* quantification of intracerebral GABA by single-voxel ^1^H-MRS–How reproducible are the results?. Eur. J. Radiol..

[64] Kondo HM, Nomura M, Kashino M (2015). Different roles of COMT and HTR2A genotypes in working memory subprocesses. PLoS One.

