# Supplementary material for: Excitation-inhibition balance and auditory multistable perception are correlated with autistic traits and schizotypy in a non-clinical population
Source: Sci Rep. 2020 May 18;10:8171. doi: 10.1038/s41598-020-65126-6 (PMC7234986; doi:10.1038/s41598-020-65126-6)
Supplement: Supplementary file 1 — Supplementary Information. [file 41598_2020_65126_MOESM1_ESM.pdf]

Supplementary Information

Excitation-inhibition balance and auditory multistable perception are correlated with autistic traits and schizotypy in a non-clinical population

Hirohito M. Kondo[1, 2, *] and I-Fan Lin[3,4]

[1]School of Psychology, Chukyo University, Nagoya, Aichi 466-8666, Japan

[2]Human Information Science Laboratory, NTT Communication Science Laboratories, NTT Corporation, Atsugi, Kanagawa 243-0198, Japan

[3]Department of Occupational Medicine, Shuang Ho Hospital, New Taipei City 235, Taiwan

[4]Department of Medicine, Taipei Medical University, Taipei 110, Taiwan

Measure	Variable	AQ Total	SPQ Total	Auditory streaming	Verbal transformations
Switch numbers	Auditory streaming	*r* = -0.060<br>*p* = 0.774	*r* = 0.147<br>*p* = 0.484	N/A	N/A
	Verbal transformations	*r* = -0.356<br>*p* = 0.039	*r* = -0.339<br>*p* = 0.050	N/A	N/A
Glx/GABA ratio	Auditory cortex	***r* = 0.468**<br>***p* = 0.008**	*r* = 0.437<br>*p* = 0.014	*r* = 0.180<br>*p* = 0.399	*r* = -0.055<br>*p* = 0.769
	Inferior frontal cortex	*r* = 0.251<br>*p* = 0.198	*r* = 0.233<br>*p* = 0.232	*r* = 0.206<br>*p* = 0.369	*r* = 0.024<br>*p* = 0.902
	Prefrontal cortex	*r* = -0.096<br>*p* = 0.612	*r* = 0.097<br>*p* = 0.612	*r* = 0.120<br>*p* = 0.577	*r* = -0.017<br>*p* = 0.927
	Anterior cingulate cortex	*r* = -0.140<br>*p* = 0.496	*r* = -0.135<br>*p* = 0.512	*r* = 0.026<br>*p* = 0.915	*r* = 0.010<br>*p* = 0.961

Supplementary Table 1. Correlations between AQ/SPQ scores, numbers of perceptual switches and Glx/GABA ratio in voxels of interest. Values indicated in bold are significant after false discovery rate (FDR) correction.

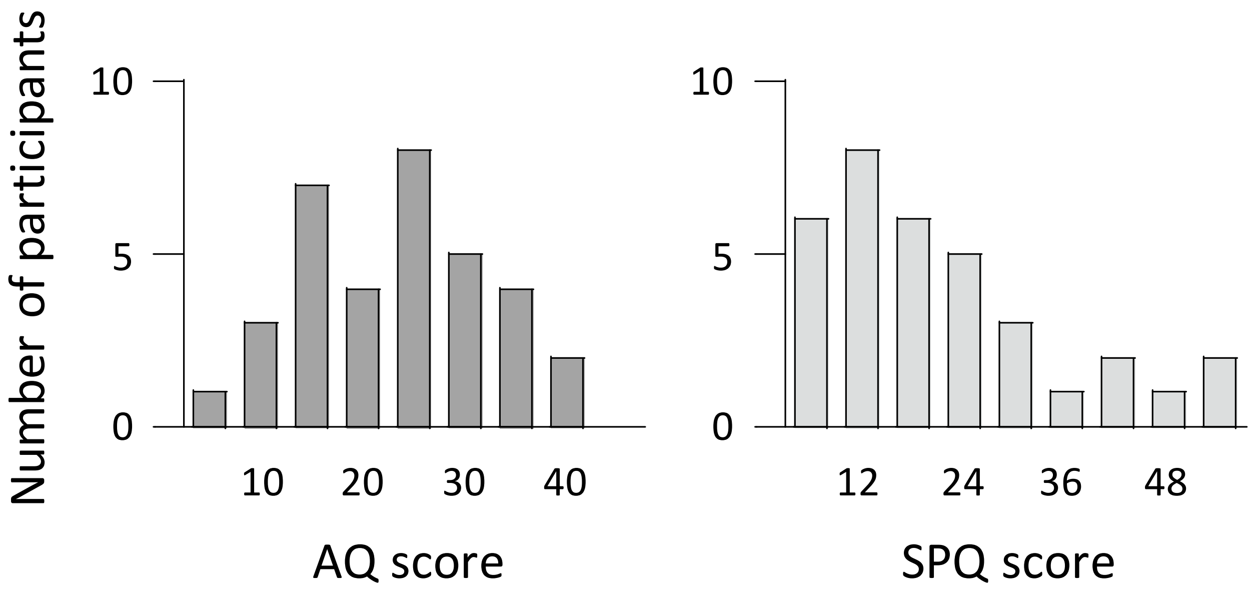

Supplementary Figure 1. Distribution of AQ and SPQ scores (*N* = 34).

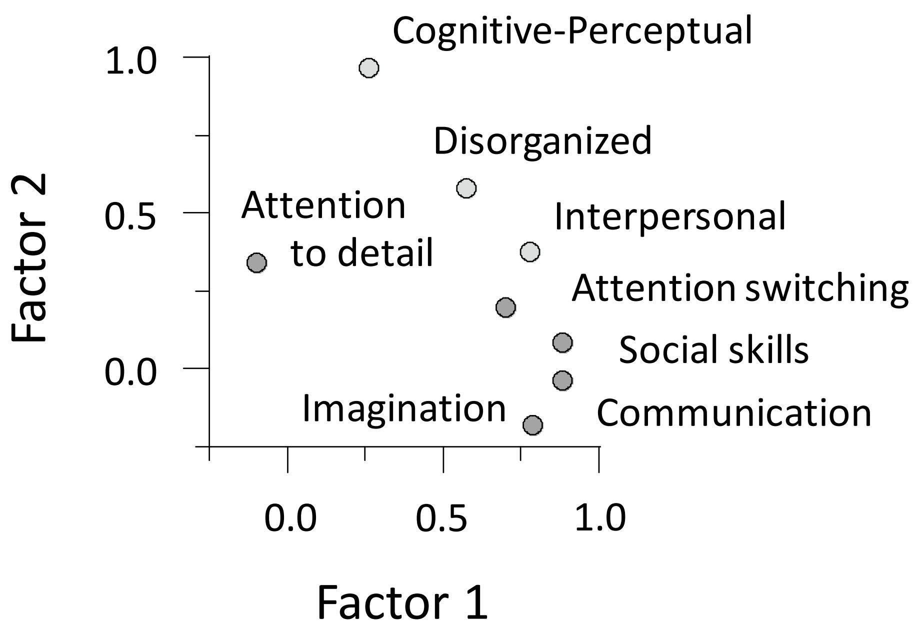

Supplementary Figure 2. Results of factor analysis. AQ and SPQ subscores for each participant were used as observed variables. Two factors were extracted using maximum likelihood method and then subjected to varimax rotation. Circles are plotted on basis of factor loadings for AQ and SPQ subscales.
